# Gender and Overdose Risk Factors Among Clients Entering Residential Treatment for Opioid Use

**DOI:** 10.1111/dar.70004

**Published:** 2025-07-07

**Authors:** Chloe J. Haynes, Alison K. Beck, Peter J. Kelly, Mei Lin Lee, Robert Stirling, Suzie Hudson, Laura Robinson, Michele Campbell, Carolyn Stubley, Briony Larance

**Affiliations:** ^1^ School of Psychology University of Wollongong Wollongong Australia; ^2^ Network of Alcohol and Other Drug Agencies Sydney Australia; ^3^ Drug Policy Modelling Program, Social Policy Research Centre UNSW Sydney Sydney Australia; ^4^ Centre for Alcohol and Other Drugs NSW Ministry of Health Sydney Australia; ^5^ National Drug and Alcohol Research Centre UNSW Sydney Sydney Australia; ^6^ School of Psychology University of Queensland Brisbane Australia; ^7^ We Help Ourselves (WHOS) Sydney Australia

**Keywords:** latent class analysis, opioid use, overdose, polysubstance use, residential treatment

## Abstract

**Introduction:**

The period post‐residential treatment for opioid use is associated with heightened risk of overdose. The current study aims to: (i) describe characteristics of men and women attending residential treatment for primary opioid use; (ii) identify classes of clients based on primary opioid and other substance/s of concern and (iii) identify relationships between substance use profile and sociodemographic risk factors for opioid overdose, including differences by gender.

**Methods:**

Data from 2994 clients (29.6% women) attending residential treatment for opioid use in the non‐government sector in New South Wales, Australia, were included in the analysis. Descriptive and chi‐square statistics analysed demographic, clinical, substance use and service use characteristics of participants. Participants were grouped using latent class analysis based on their primary opioid of concern and other substance/s of concern. Multinomial logistic regression examined the relationship between latent classes and sociodemographic risk factors for overdose, including interactions with gender.

**Results:**

Men and women presented to residential treatment with different demographic, clinical, substance use and service use characteristics. A five‐class model of polysubstance use was identified: *heroin + lower polysubstance use* (52.3%), *heroin + polysubstance use* (22.2%), *pharmaceutical + lower polysubstance use* (10.1%)*, pharmaceutical + polysubstance use* (6.7%) and *OAT + polysubstance use* (8.7%). There were some associations between sociodemographic risk factors and class membership, though limited interactions between sociodemographic risk and gender.

**Discussion and Conclusions:**

Effective overdose prevention and harm reduction strategies during and post‐residential treatment need to consider individuals' complex and unique accumulation of risk.

**Summary**

- Men and women present to residential treatment for opioid use with different sociodemographic profiles.
- Polysubstance use is common among people attending residential treatment for opioid use.
- Important to consider interactions between sociodemographic risk and polysubstance use.
- Harm reduction and post‐treatment support in basic life domains are essential for all clients.
- Early identification of overdose risk important for treatment planning and provision of individualised post‐treatment support.

## 1 Introduction

Responding to the opioid overdose crisis is an important clinical and policy priority, with more than 100,000 people dying of opioid overdose every year [1]. In line with the United Nations Office on Drugs and Crime [2] Opioid strategy, strengthening and supporting opioid treatment is an important aspect of addressing this crisis. The ‘gold standard’ treatment for opioid use disorder is opioid agonist treatment (OAT; i.e., methadone, buprenorphine) in combination with psychosocial support [3, 4]. However, residential treatment is often viewed as effective and high‐level care for alcohol and other drug (AOD) use [5] and is associated with significant improvements substance use and psychosocial outcomes [6].

Residential treatment emphasises comprehensive care, often integrating counselling, harm reduction education and practical skill development to reduce substance use and improve general functioning [7]. Effective tailoring of this care requires an appreciation of the context that brings each client to treatment [6]. This includes understanding the interacting individual, interpersonal, community and societal factors that impact opioid use and related harms [8]. Clients accessing outpatient treatment are more likely to return to substance use at 12 months following treatment than clients accessing inpatient/residential services [9]. However, research demonstrates that any period immediately following an opioid treatment episode is associated with an increased risk of fatal overdose [10, 11]. In both inpatient and outpatient opioid treatment settings, this risk is exacerbated by the presence of co‐occurring sociodemographic risk factors such as being middle aged (35–55) or older (55+) [12, 13], homelessness/unstable accommodation [14, 15], lower socioeconomic status [13], living alone (as 78% of drug‐related deaths from 1997 to 2020 occurred in the home) [12], having spent time in prison [16, 17] or having other criminal justice involvement [18], experiencing mental health conditions or higher psychological distress [13, 19] and injecting drug use or unsafe injecting practices [14, 20]. This highlights the importance of early identification of risk factors to effectively tailor treatment to manage risks during a client's treatment episode, as well as the integration of appropriate supports in the post‐treatment period. Consumer representatives have specifically voiced their desire for improved supports post‐residential treatment, such as additional/improved support regarding housing, employment and mental health [6].

A client's pattern of substance use also provides important insights regarding their risk of post‐treatment substance use or opioid overdose (specifically respiratory depression, unconsciousness or death). Research has consistently demonstrated that people who use opioids also commonly use other substances [21, 22], including alcohol, cannabis, stimulants and/or benzodiazepines [23]. Combining opioids with other substances, particularly, other depressants, increases the risk of overdose due respiratory depression [24]. This indicates the importance of comprehensive risk assessment and discharge planning to adequately address each client's unique substance use profile, inclusive of primary and other substance/s of concern. In addition, residential treatment for opioid use may or may not provide concurrent pharmacological treatment (e.g., methadone, buprenorphine). Residential treatments may also require clients to complete withdrawal before entering treatment, resulting in many clients who are opioid naïve upon treatment completion or exit [25]. This opioid naivety increases the risk of post‐treatment overdose if clients return to opioid (and/or other substance) use [26], particularly, in the context of additional sociodemographic risk factors.

It is also important that treatment planning and provision consider the influence of gender when assessing overdose risk. Though men have a higher rate of fatal opioid overdose compared to women [12, 13], research has demonstrated that the profile of women who present to treatment for opioid use is different from men in terms of demographics, substance use, service utilisation and the types and severity of associated problems. For example, women who use opioids are often younger than men [27, 28, 29, 30] and are more likely to be unemployed [28, 30, 31, 32, 33]. Women also tend to have fewer years of opioid use prior to seeking treatment [29, 34, 35, 36] and have more physical/medical problems, greater psychiatric comorbidity and higher rates of suicidality [28, 29, 30, 31, 35]. While most of this research is focused on cisgender men and women, it highlights the importance of considering gendered experiences of opioid use, treatment and post‐treatment overdose risk. The lack of gender representation in the literature is also consistent with the emerging nature of gendered considerations of substance use and the limited number of non‐binary and transgender clients who access mainstream residential substance use treatment [37], though this represents an important area for future research.

In order to maximise treatment outcomes and manage overdose risk for people who use opioids, it is important to consider the entirety of a person's substance use as well as the relationship between gender and sociodemographic overdose risk factors [38, 39]. This study will examine the co‐occurrence of overdose risk factors among Australian opioid users accessing residential treatment, particularly, how these risks differ by gender. The current study aims to: (i) describe demographic, clinical, substance use and service utilisation characteristics of men and women attending residential treatment for primary opioid use in New South Wales, Australia; (ii) identify unique classes of clients at treatment entry based on primary opioid of concern and other substance/s of concern; (ii) identify relationships between class membership and sociodemographic risk factors for opioid overdose; and (iv) explore how these risks differ by gender. This knowledge is important for overdose prevention and harm reduction efforts, including the importance of tailoring client support both during and post‐treatment.

## 2 Methods

### 2.1 Dataset and Study Design

The Network of Alcohol and Other Drug Agencies (NADA) is a peak body for non‐government AOD treatment services, representing over 80 organisations that are primarily based in New South Wales, with some services in Queensland and the Australian Capital Territory. Given the diversity of NADA members, models of care applied in the delivery of services, including the duration of treatment, may vary. NADA manages the NADABase, which is a system for client data collection and reporting. This data collection is governed by the NADA Data Management Policy, available on request. The NADABase contains client data encompassing the National Minimum Data Set (NMDS) and outcome measures (see Section 2.3). Data are self‐reported by clients at treatment entry (as well as 30‐, 60‐ and 90‐days post‐treatment entry for outcome data). Data collection and uploading is facilitated by staff. Member services include residential and community services that are primarily publicly funded by state or federal governments. Privately funded services or services receiving philanthropic funding are not mandated to upload their data to the NADABase. Consent to provide data is obtained at the service level.

For the current study, clients' treatment entry data from their current or most recent treatment episode between January 2012 and August 2023 was used in a cross‐sectional analysis. This study followed the Strengthening the Reporting of Observational Studies in Epidemiology (STROBE) guidelines for cross‐sectional research (see Appendix A). The study received approval from the University of Wollongong Human Research Ethics Committee (HE12/207) and was endorsed by the Community Mental Health, Drug and Alcohol Research Network (CMHDARN) Research Ethics Consultation Committee.

### 2.2 Participants

**FIGURE 1:**
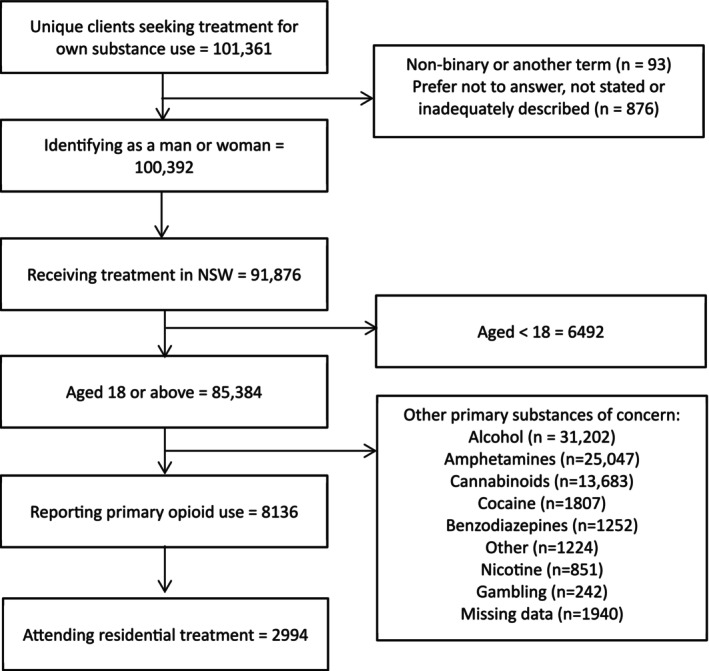
Identification and selection of NADAbase clients for participation in this study.

Participants were drawn from the NADAbase and included in analysis if they: (i) were aged 18 or over; (ii) reported gender as a man or woman; (iii) were attending residential treatment for their own substance use and (iv) identified an opioid as their primary substance of concern. Unique client's current or most recent treatment data were identified in the dataset. The process of identifying participants for the current study is shown in a flow chart (see Figure 1).

A total of 2994 participants that reported primary opioid use at presentation to residential treatment were included in the current study, including 885 (29.6%) women. Participants had a mean age of 37.29 years (SD = 9.14 years). The majority were born in Australia (89.1%) and reported heroin use at treatment entry (74.5%).

### 2.3 Measures

The NADAbase Data Dictionary [40] describes the data collected for the NMDS and Client Outcome Measurement System. The NMDS collects data across a range of service, demographic and substance use characteristics. For the current study, specific demographic (age, country of birth, sexuality, accommodation, living arrangements, source of income, location of usual residence), substance use (primary opioid of concern, other substance/s of concern, lifetime injecting use, prior overdoses) and service‐related (source of referral, main service accessed, location of service) characteristics were recoded and used in analysis (see Appendix B). Data regarding opioid and other substance/s of concern followed the Australian Standard Classification of Drugs of Concern [41] and were analysed at the ‘base level’ unit for primary opioid of concern (e.g., heroin, fentanyl, oxycodone) and the ‘narrow group’ unit for other substances of concern (e.g., alcohol, amphetamines, cannabinoids). Service‐identifier codes were also provided by NADA to identify participants accessing residential services that prescribe OAT. Additional measures collected as part of the Client Outcome Measurement System are described below.

### 2.4 Substance Dependence Severity Scale (SDS; [42])

The SDS is a five‐item measure which assess the extent of psychological dependence for a particular substance of concern. Items are rated from 0 (‘never’) to 3 (‘often’), with item scores summed to produce a total score reflecting severity of dependence. Cut off scores for dependence are based on the substance of concern reported by the client, for example, > 5 for heroin [43, 44]. The SDS has been validated for use in both specialist and non‐specialist substance use treatment settings [43]. In the current study, the SDS was used to characterise participant's degree of substance dependence at treatment entry.

### 2.5 Kessler Psychological Distress Scale (K10+; [45])

The K10+ is a 10‐item measure assessing psychological distress, with items rated on a 5‐point Likert Scale. Item scores are summed to produce a total score between 10 and 50, with higher scores reflecting greater psychological distress, categorised into low (10–15), moderate (16–21), high (22–29) and very high (30–50) [46]. The K10+ has been validated for use among individuals who use substances [47, 48]. The K10+ was used to characterise participants' degree of psychological distress at treatment entry.

### 2.6 World Health Organisation 8—Quality of Life (WHO 8‐QoL; [49])

The WHO 8‐QoL is an 8‐item measure that assess quality of life across four domains (physical health, psychological wellbeing, social relationships and environment). Each item is rated from 1 (‘not at all’) to 5 (‘completely’) and scores are summed to produce a total score from 8 to 40, with higher scores indicating better quality of life. The WHO 8‐QoL has been validated cross‐culturally [50] and was used to characterise participant's quality of life at treatment entry.

### 2.7 Statistical Analysis

#### 2.7.1 Demographic Analysis

Analysis was conducted in IBM SPSS Statistics (Version 29.0). Variables were recoded for analysis to aid in presentation/interpretation of results (see Appendix B). Descriptive statistics were used to explore the demographic, clinical, substance use and service use characteristics of clients at treatment entry. Chi‐square tests were used to assess gender differences in each variable and z tests with Bonferroni correction were used to identify significantly different proportions of men and women within each level of each variable at *p* < 0.05.

#### 2.7.2 Coding of Risk Factors

Demographic and substance use variables known to be associated with higher overdose risk (e.g., middle or older age, unstable accommodation, living alone, criminal justice involvement, injecting drug use) were recoded into overdose risk factors, reflecting ‘higher’ vs. ‘lower’ risk (see Appendix C). For the purposes of analysis and in an attempt not to under‐ or over‐estimate risk, responses of ‘other’ or ‘unknown’ (i.e., not stated, inadequately described, not collected) were retained as separate categories. Service‐ and clinician‐level procedures may explain the varying amounts of missing data, with some items (e.g., sexuality, prior overdose) not mandated or reliably collected/reported across services (i.e., as part of NMDS). Although psychological distress (K10), severity of dependence (SDS) and quality of life (WHO 8‐QoL) are relevant to overdose risk, they were excluded from subsequent analyses due to substantial missing data, as not all services collect and report these data routinely (or use alternative measures at service‐level). Chi‐square tests were used to assess differences in the proportion of men and women identified as ‘higher risk’ for each risk factor. Due to small numbers of participants reporting primary fentanyl use, primary fentanyl was combined with primary pharmaceutical opioid use for all following analyses.

### 2.8 Latent Class Analysis (LCA)

MPlus was used for LCA [51, 52]. LCA was used to determine latent classes of polysubstance use based on nine binary variables: primary opioid of concern (heroin, pharmaceutical opioid, methadone/buprenorphine) and other substance/s of concern (another opioid, alcohol, sedatives, cannabis, stimulants, other substance). Consistent with Weller et al. [53], multiple fit statistics were used to determine the optimal class solution, including: (a) log‐likelihood; (b) Akaike Information Criterion; (c) Bayesian Information Criterion (BIC); (d) sample‐size adjusted Bayesian Information Criterion (SABIC); (e) Vuong–Lo–Mendell–Rubin adjusted likelihood ratio test (VLMR‐LRT) and (f) bootstrapped likelihood ratio test (B‐LRT). For log‐likelihood, Akaike Information Criterion, BIC and sample‐size adjusted BIC, lower values indicate better model fit [53]. For VLMR‐LRT and B‐LRT, *p* values greater than 0.05 indicate that the current class model is no better than a model with one less class [54]. Diagnostic criteria of entropy and average latent class posterior probabilities were also examined, with values above 0.80 considered acceptable [53]. Class sizes and interpretability were also considered in selecting an optimal solution [53].

#### 2.8.1 Multinomial Logistic Regression

Consistent with the three‐step approach [55, 56], once an optimal solution was determined and participants were assigned to their latent class, sociodemographic overdose risk factors, as well as the interaction between these risk factors and gender, were added as covariates in a multinomial logistic regression model predicting class membership. All predictors were entered in a single step. Odds ratios, confidence intervals and statistical significance are reported.

## 3 Results

### 3.1 Gender Differences in Demographic, Clinical, Substance Use and Service Utilisation Characteristics

Proportional differences between men and women (% reported for men and women respectively) were observed across all demographic characteristics (Table 1). A higher proportion of women were aged 18–29 years (18.3% vs. 26.4%), born in Australia (88.1% vs. 91.4%), identified as LGBTQIA+ (0.5% vs. 2.3%), lived alone (29.9% vs. 37.1%) or with dependent children (10.1% vs. 14.7%) and received permanent government benefits (18.3% vs. 25.2%). Compared to women, a higher proportion of men were aged 40–49 (28.4% vs. 23.7%) or 50–59 (9.9% vs. 6.7%), born in Asia (5.1% vs. 2.7%), lived in prison/detention centres (5.5% vs. 2.9%) or with parents/relatives/friends (41.2% vs. 34.1%) and were either in full time employment (6.0% vs. 3.7%) or had no income (5.3% vs. 2.8%). A higher proportion of women reported primary fentanyl use (1.4% vs. 2.6%) and other stimulant use (25.5% vs. 29.4%). Women also had higher average psychological distress at treatment entry compared to men (*M* = 22.98 vs. *M* = 25.68). In terms of service use characteristics, a higher proportion of women received referrals for treatment via non‐residential (4.2% vs. 6.2%), other non‐health (1.0% vs. 2.1%) or family and child protective services (0.2% vs. 3.4%), whereas more men received referrals through the police or the criminal justice system (CJS) (23.0% vs. 15.4%). Despite a higher proportion of women living in non‐metropolitan areas (e.g., remote areas; 1.7% vs. 4.1%), more women were accessing treatments located in metropolitan areas (63.2% vs. 68.5%). Compared to men, a significantly higher proportion of women were accessing residential treatments that prescribe OAT (18.0% vs. 21.6%).

**TABLE 1:** Gender differences in demographic, clinical, substance use and service use characteristics at intake to residential treatment for primary opioid use (*N* = 2294).

Characteristic	Total sample (*N* = 2994)		Men (*n* = 2109)		Women (*n* = 885)		Difference between men and women	
	*n*	%	*n*	%	*n*	%	*X* ^2^	*p*
*Demographic characteristics*								
Age^a^							**32.688**	**< 0.001**
18–29 years	620	20.7	386	18.3	234	**26.4** ^b^		
30–39 years	1257	42.0	885	42.9	372	42.0		
40–49 years	808	27.0	598	**28.4** ^b^	210	23.7		
50–59 years	267	8.9	208	**9.9** ^b^	59	6.7		
60+ years	42	1.4	32	1.5	10	1.1		
Country of birth^a^							**18.637**	**0.005**
Australia	2668	89.1	1859	88.1	809	**91.4** ^b^		
Asia	132	4.4	108	**5.1** ^b^	24	2.7		
Europe	83	2.8	58	2.8 1.4	25 15	2.8 1.7		
New Zealand	44	1.6	29					
Other Oceania^c^	18	0.6	12	0.6	6	0.7		
Other	44	1.5	40	**1.9** ^b^	4	0.5		
Unknown	5	0.2	3	0.1	2	0.2		
Sexuality							**23.794**	**< 0.001**
Straight/heterosexual	504	16.8	339	16.1	165	18.6		
LGBTQIA+	30	1.0	10	0.5	20	**2.3** ^b^		
Not stated/inadequately described	2454	82.0	1756	**83.3** ^b^	698	78.9		
Prefer not to answer	6	0.2	4	0.2	2	0.2		
Accommodation^a^							**11.170**	**0.048**
Stable accommodation	1942	64.9	1354	64.2	588	66.4		
Unstable/temporary accommodation^d^	289	9.7	212	10.1	77	8.7		
Prison/detention centre	141	4.7	115	**5.5** ^b^	26	2.9		
Homeless/no usual residence	251	8.4	170	8.1	81	9.2		
Other	76	2.5	54	2.6	22	2.5		
Unknown	295	9.9	204	9.7	91	10.3		
Living arrangements^a^							**19.581**	**< 0.001**
Alone	959	32.0	631	29.9	328	**37.1** ^b^		
Spouse or partner	414	13.8	293	13.9	121	13.7		
Parents/relatives/friends	1171	39.1	869	**41.2** ^b^	302	34.1		
Other	321	10.7	231	11.0	90	10.2		
Unknown	129	4.3	85	4.0	44	5.0		
Living with dependent children (yes)	342	11.4	212	10.1	130	**14.7** ^b^	**13.428**	**< 0.001**
Source of income^a^							**30.079**	**< 0.001**
Full or part time employment	160	5.3	127	**6.0** ^b^	33	3.7		
Temporary government benefits^e^	1989	66.4	1414	67.0	575	65.0		
Permanent government benefits^f^	610	20.4	387	18.3	223	**25.2** ^b^		
No income	137	4.6	112	**5.3** ^b^	25	2.8		
Other	54	1.8	40	1.9	14	1.6		
Unknown	44	1.5	29	1.4	15	1.7		
Location of usual residence^a^							**28.178**	**< 0.001**
Major cities	2022	67.5	1430	67.8	592	66.9		
Regional	786	26.3	545	25.8	241	27.2		
Remote	72	2.4	36	1.7	36	**4.1** ^b^		
Unknown	114	3.8	98	**4.6** ^b^	16	1.8		
*Substance use characteristics*								
Primary opioid of concern^a^							10.274	0.174
Heroin	2230	74.5	1588	75.3	642	72.5		
Oxycodone	183	6.1	129	6.1	54	6.1		
Codeine	85	2.8	53	2.5	32	3.6		
Morphine	57	1.9	37	1.8	20	2.3		
Fentanyl	53	1.8	30	1.4	23	**2.6** ^b^		
Methadone	174	5.8	121	5.7	53	6.0		
Buprenorphine	85	2.8	64	3.9	21	2.4		
Other opioid analgesic^g^	127	4.2	87	4.1	40	5.4		
Other substance(s) of concern^a^, ^h^								
Another opioid	544	18.2	384	18.2	160	18.1	0.001	0.934
Alcohol	383	12.8	284	13.5	99	11.1	2.904	0.088
Benzodiazepines or sedatives^i^	386	12.9	272	12.9	114	12.9	0.000	0.991
Cannabinoids	600	20.0	417	19.8	183	20.7	0.319	0.572
Stimulants^j^	798	26.7	538	25.5	260	**29.4** ^b^	**4.773**	**0.029**
Other substance^k^	452	15.1	327	15.5	125	14.1	0.927	0.336
Lifetime injecting use^a^							**21.785**	**< 0.001**
Yes	2392	79.9	1701	80.7	691	78.1		
No	460	15.4	331	15.7	129	14.6		
Not stated/inadequately described	112	3.7	57	2.7	55	**6.2** ^b^		
Not collected	30	1.0	20	0.9	10	1.1		
Prior overdose(s)							2.248	0.325
None	1117	37.3	794	37.6	323	36.5		
One or more	109	3.6	70	4.4	39	4.4		
Not collected	1768	59.1	1245	59.0	523	59.1		
*Treatment characteristics*								
Source of referral^a^							**92.415**	**< 0.001**
Self	1365	45.6	946	44.9	419	47.3		
Family member/friend	210	7.0	160	7.6	50	5.6		
Medical professional/hospital	135	4.5	86	4.1	49	5.5		
Residential community care unit/agency	285	9.5	210	10.0	75	8.5		
Non‐residential community care unit/agency	143	4.8	88	4.2	55	**6.2** ^b^		
Other non‐health service	41	1.4	22	1.0	19	**2.1** ^b^		
Police/court diversion or justice system	621	20.7	485	**23.0** ^b^	136	15.4		
Family and child protective services	35	1.2	< 10	0.2	30	**3.4** ^b^		
Other^l^	114	3.8	80	3.8	34	3.8		
Not stated/inadequately described	45	1.5	27	1.3	18	2.0		
Main service accessed^a^							8.101	0.151
Counselling	29	1.9	19	0.9	10	1.1		
Withdrawal management	427	14.3	286	13.6	141	15.9		
Rehabilitation	1905	63.6	1163	64.6	542	61.2		
Support and case management	120	4.0	83	3.9	37	4.2		
Assessment only	458	15.3	326	15.5	132	14.9		
Other^m^	55	1.8	32	1.5	23	**2.6** ^b^		
Accessing service that prescribes OAT	570	19.0	379	18.0	**191**.	**21.6** ^b^	**5.275**	**0.022**
Location of service^a^							**7.789**	**0.020**
Major cities	1938	64.7	1332	63.2	606	**68.5** ^b^		
Regional	1043	34.8	767	36.4	275	31.2		
Remote	14	0.4	10	0.5	< 10	0.3		

Baseline COMS measure	Total		Men		Women		Difference	
	M	SD	M	SD	M	SD	*t*	*p*
Psychological distress (K10+)^n^	23.77	9.12	22.98	8.67	**25.68**	**9.88**	**−4.469**	**< 0.001**
Severity of dependence (SDS)^o^	8.59	4.20	8.46	4.12	8.94	4.42	−1.715	0.087
Quality of life (EUROHIS QoL)^p^	25.53	6.27	25.75	6.17	25.00	6.46	1.840	0.066

Abbreviations: COMS, Client Outcome Measurement System; K10, Kessler Psychological Distress Scale; OAT, opioid agonist treatment; SDS, Substance Dependence Severity Scale.

^a^ Indicates variables that are mandated/routinely collected as part of the National Minimum Data Set.

^b^ Indicates higher proportion based on z‐test (with Bonferroni correction) comparing proportion (%) of males and females within each level of each variable, *p* < 0.05 (in bold).

^c^ *Other Oceania* includes Micronesia, Melanesia and Polynesia.

^d^ *Unstable/temporary accommodation* includes boarding house, hostel or supported accommodation, psychiatric hospital, alcohol and other drug treatment centre, shelter/refuge and caravan on a serviced site.

^e^ *Temporary government benefits* includes Student Allowance, Newstart Allowance, Youth Training Allowance, Sickness Allowance, Special Benefit, Widow Allowance or Mature Age Allowance (granted on or AFTER 1 July 1996).

^f^ *Permanent government benefits* include Age Pension, Disability Support Pension, Disability Wage Supplement, Carer Pension, Wife Pension, Widow Pension (Class B, Bereavement Allowance), Mature Age Allowance (granted before 1 July 1996), Mature Age Partner Allowance, Sole Parent Pension or Veterans Affairs Benefit.

^g^ *Other opioid analgesic* includes levomethadyl acetate hydrochloride, meperidine analogues, pethidine, tramadol and any other pharmaceutical opioids or organic, semisynthetic or synthetic opiate analgesics not further defined.

^h^ Substance identified as any additional substance of concern.

^i^ *Benzodiazepines and sedatives* include anaesthetics, barbiturates, benzodiazepines, GHB and other sedatives or hypnotics.

^j^ *Psychostimulants* includes amphetamines, ephedra alkaloids, cocaine and methylphenidate.

^k^ *Other* includes gambling, non‐opioid analgesics, hallucinogens, caffeine, nicotine, steroids, antidepressants, antipsychotics, inhalants and ‘other’.

^l^ *Other* includes educational institution, workplace, needle and syringe programmes, medically supervised injection centres.

^m^ *Other* includes pharmacotherapy, information only, detoxification.

^n^ Only includes *n* = 1086 (men = 770, women = 316) with valid K10+ data at baseline.

^o^ Only includes *n* = 1076 (men = 764, women = 312) with valid SDS data at baseline.

^p^ Only includes *n* = 1124 (men = 785, women = 339) with valid EUROHIS QoL data at baseline.

### 3.2 Sociodemographic Risk Factors for Overdose

Compared to men, a significantly higher proportion of women were characterised as ‘higher risk’ related to living alone (29.9% vs. 37.1%), being unemployed (90.7% vs. 93.0%), living in a regional/remote area (27.5% vs. 31.3%) and reporting primary pharmaceutical (15.9% vs. 19.1%) or other stimulant use (25.5% vs. 29.4%) (Table 2). Compared to women, a significantly higher proportion of men were characterised as ‘higher risk’ related to age (i.e., being middled aged to older; 63.5% vs. 52.8%) and reporting CJS involvement (25.3% vs. 16.4%). Accessing a residential service that prescribes OAT was also associated with being aged 35+ years (adjusted odds ratio [AOR] = 1.262, *p* = 0.028), living in unstable accommodation (AOR = 0.53, *p* < 0.001) or alone (AOR = 1.461, *p* < 0.001), being unemployed (AOR = 2.533, *p* < 0.001), having criminal justice involvement (AOR = 0.354, *p* < 0.001) and reporting high‐very high baseline psychological distress (AOR = 4.655, *p* < 0.001)

**TABLE 2:** Proportions of men and women classified as ‘*higher risk*’ vs. *‘lower risk’, ‘other’* and *‘unknown’* for each sociodemographic and substance use risk factor.

Risk factor	Total sample (*N* = 2994)		Men (*n* = 2109)		Women (*n* = 885)		Difference between men and women
	*n*	%	*n*	%	*n*	%	*X* ^2^ *, p*
Age							**24.493, *p* < 0.001**
Younger (18–34)	1209	40.4	791	37.5	418	**47.2** ^a^	
Middle‐aged to older (35+)^b^	1785	59.6	1318	**63.5** ^a^	467	52.8	
Accommodation							2.841, *p* = 0.417
Stable	1942	64.9	1354	64.2	588	66.4	
Unstable^b^	681	22.7	497	23.6	184	20.8	
Other	76	2.5	54	2.6	22	2.5	
Unknown	295	9.9	204	9.7	91	10.3	
Living arrangements							**17.845, *p* < 0.001**
With others	1585	52.9	1162	**55.1** ^a^	423	47.8	
Alone^b^	959	32.0	631	29.9	328	**37.1** ^a^	
Other	321	10.7	231	11.0	90	10.2	
Unknown	129	4.3	85	4.0	44	5.0	
Employment							7.267, *p* = 0.064
Employed	160	5.4	127	**6.0** ^a^	33	3.7	
Unemployed^b^	2736	91.4	1913	90.7	823	**93.0** ^a^	
Other	54	1.8	40	1.9	14	1.6	
Unknown	44	1.5	29	1.4	15	1.7	
Criminal justice involvement							**34.852, *p* < 0.001**
No	2293	76.6	1566	74.3	727	**82.1** ^a^	
Yes (living in prison OR CJS referral)^b^	678	22.6	533	**25.3** ^a^	145	16.4	
Unknown (other/unknown for both)	23	0.8	10	0.5	10	**1.5** ^a^	
Region							**16.332, *p* < 0.001**
Major city	2022	67.5	1430	67.8	592	66.9	
Regional/remote^b^	858	28.7	581	27.5	277	**31.3** ^a^	
Unknown	114	3.8	98	**4.6** ^a^	16	1.8	
Recent injecting drug use							**19.821, *p* < 0.001**
No	1130	37.7	817	38.7	313	35.4	
Yes (within last 3 months)^b^	1722	57.5	1215	57.6	507	57.3	
Unknown	142	4.7	77	3.7	65	**7.3** ^a^	
Primary opioid of concern							
Heroin	2230	74.5	1588	75.3	642	72.5	2.488, *p* = 1.115
Pharmaceutical opioid^c^	505	16.9	336	15.9	169	**19.1** ^a^	4.425, *p* = 0.035
Methadone/buprenorphine	259	8.7	185	8.8	74	8.4	0.133, *p* = 0.716
Other substance(s) of concern							
Another opioid	544	18.2	384	18.2	160	18.1	0.001, *p* = 0.934
Alcohol	383	12.8	284	13.5	99	11.1	2.904, *p* = 0.088
Benzodiazepines or sedatives	386	12.9	272	12.9	114	12.9	0.000, *p* = 0.991
Cannabinoids	600	20.0	417	19.8	183	20.7	0.319, *p* = 0.572
Stimulants	798	26.7	538	25.5	260	**29.4** ^a^	**4.773, *p* = 0.029**
Other substance	452	15.1	327	15.5	125	14.1	0.927, *p* = 0.336

Abbreviation: CJS, criminal justice system.

^a^ Higher proportion based on *z* test (with Bonferroni correction) comparing proportion (%) of males and females within each level of each variable, *p* < 0.05 (in bold).

^b^ ‘higher risk’ category.

^c^ Includes oxycodone, morphine, fentanyl, levomethadyl acetate hydrochloride, meperidine analogues, pethidine, tramadol and any other pharmaceutical opioids or organic, semisynthetic or synthetic opiate analgesics not further defined.

### 3.3 LCA Based on Patterns of Opioid and Other Substance Use and Associations With Sociodemographic Risk Factors

Based on patterns of primary opioid and other substance use, a five‐class model was determined as the optimal solution. Though LRT statistics demonstrated that 6 classes significantly improved the model compared to five classes, there was little change in BIC for the six‐class model and a reduction in diagnostic accuracy (Table 3). Additionally, class sizes and interpretability supported five classes as the optimal solution.

**TABLE 3:** Latent class analysis model fit and diagnostic criteria.

Model	AIC	BIC	SABIC	Smallest class count			Entropy	ALCPP	VLMR‐LRT	
				Class	*n*	%			Value	*p*
2 class	21,728.39	21,842.48	21,782.11	Class 1	764	25.5	1.00	1.00	−12,161.44	< 0.001
3 class	20,765.41	20,939.54	20,847.39	Class 3	259	8.7	1.00	1.00	−10,845.20	< 0.001
4 class	20,343.06	20,577.23	20,453.31	Class 2	259	8.7	0.81	0.92	−10,353.70	< 0.001
5 class	20,255.48	20,549.69	20,394.00	Class 1	202	6.7	0.80	0.88	−10,132.53	< 0.001
6 class	20,215.89	20,570.15	20,382.68	Class 3	65	2.2	0.81	0.86	−10,078.84	0.003

Abbreviations: AIC, Akaike Information Criterion; ALCPP, average latent class posterior probability; BIC, Bayesian Information Criterion; SABIC, sample‐size adjusted Bayesian Information Criterion; VLMR‐LRT, Vuong–Lo–Mendell–Rubin adjusted likelihood ratio test.

**FIGURE 2:**
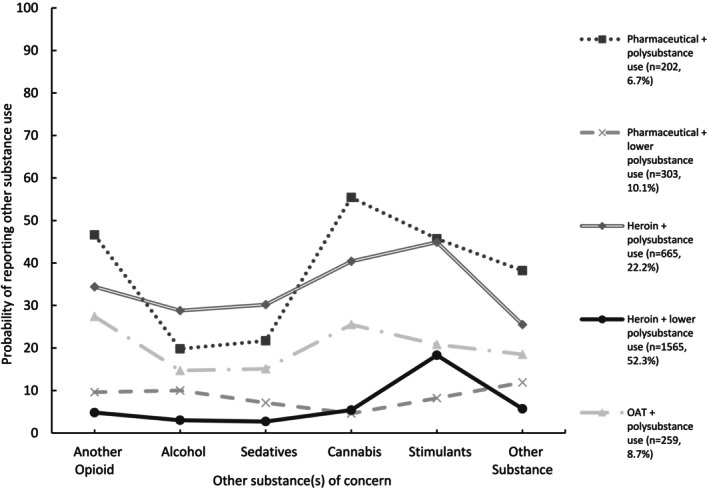
Probability of reporting other substance use by class (%).

The largest class was the ‘heroin + lower polysubstance use’ class (*n* = 1565, 52.3%). This class had high probability of primary heroin use and low probability of other substance use, except for other stimulant use. In contrast, the ‘heroin + polysubstance use’ class (*n* = 665, 22.2%) had high probability of primary heroin use, as well as high probability of other opioid, stimulant and cannabis use. This class also had the highest probability across all classes of alcohol and sedative use. The ‘pharmaceutical + polysubstance use’ class (*n* = 202, 6.7%) had high probability of primary pharmaceutical opioid use, as well as the highest probabilities across all classes of cannabis, stimulant, other opioid and other substance use. In contrast, the ‘pharmaceutical + lower polysubstance use’ class (*n* = 303, 10.1%) showed high probability of primary pharmaceutical opioid use and lower probability of other substance use across all substance categories. Finally, the ‘OAT + polysubstance use’ class (*n* = 259, 8.7%) showed high probability of primary methadone/buprenorphine use, as well as probabilities of other substance use ranging from 14.7% for alcohol to 27.4% for other opioid use (Figure 2 and Appendix D).

A multinomial logistic regression was conducted predicting class membership based on: (1) individual sociodemographic overdose risk factors, including gender and (2) interactions between sociodemographic risk factors and gender. All predictors were entered in a single step, with the *heroin + lower polysubstance use* class as the referent class (Table 4).

**TABLE 4:** Multinomial logistic regression predicting latent class membership based on sociodemographic overdose risk factors and interactions between risk factors and gender.

Risk factor	Latent class							
	Pharmaceutical + polysubstance use (*n* = 202, 6.7%)		Pharmaceutical + lower polysubstance use (*n* = 303, 10.1%)		Heroin + polysubstance use (*n* = 665, 22.2%)		OAT + polysubstance use (*n* = 259, 8.7%)	
	AOR	[95% CI]	AOR	[95% CI]	AOR	[95% CI]	AOR	[95% CI]
Gender (ref: male)	0.229	[0.035, 1.502]	1.048	[0.296, 3.704]	0.863	[0.243, 3.062]	0.188	[0.202, 7.763]
Age (ref: younger)	0.725	[0.494, 1.064]	0.920	[0.647, 1.308]	0.744*	[0.614, 0.975]	0.958	[0.680, 1.350]
Woman × Older	1.105	[0.495, 2.080]	1.012	[0.557, 1.835]	1.244	[0.802, 1.867]	1.902	[0.995, 3.365]
Accommodation (ref: stable)								
Unstable	1.107	[0.594, 1.769]	0.797	[0.469, 1.345]	1.345*	[1.003, 1.802]	0.917	[0.582, 1.443]
Woman × Unstable	0.676	[0.224, 2.036]	0.911	[0.366, 2.268]	0.945	[0.543, 1.646]	0.987	[0.402, 2.420]
Other	0.576	[0.127, 2.619]	0.523	[0.145, 1.883]	0.692	[0.338, 1.419]	0.254	[0.054, 1.191]
Woman × Other	—	—	0.693	[0.083, 5.597]	0.730	[0.174, 3.017]	1.215	[0.085, 17.449]
Unknown	0.490	[0.220, 1.091]	2.000**	[1.234, 3.239]	0.249**	[0.133, 0.465]	0.754	[0.417, 1.363]
Woman × Unknown	0.841	[0.178, 3.969]	0.479	[0.188, 1.220]	1.504	[0.584, 4.126]	0.830	[0.236, 2.622]
Living arrangements (ref: with others)								
Alone	1.085	[0.702, 1.676]	1.139	[0.768, 1.688]	0.061	[0.807, 1.934]	1.286	[0.877, 1.884]
Woman × Alone	0.983	[0.450, 2.147]	0.625	[0.323, 1.208]	0.768	[0.479, 1.232]	0.468*	[0.229, 0.956]
Other	0.149*	[0.034, 0.651]	0.394*	[0.166, 0.938]	1.201	[0.811, 1.778]	1.006	[0.525, 1.928]
Woman × Other	3.087	[0.317, 30.068]	2.373	[0.656, 8.584]	0.464	[0.207, 1.043]	0.907	[0.264, 3.111]
Unknown	0.154	[0.019, 1.280]	0.472	[0.160, 1.393]	0.751	[0.344. 1.638]	1.149	[0.582, 3.462]
Woman × Unknown	16.561**	[1.043, 262.994]	1.594	[0.296, 8.584]	0.801	[0.197, 3.263]	0.719	[0.157, 3.288]
Employment (ref: employed)								
Unemployed	1.300	[0.586, 2.883]	0.691	[0.399, 1.196]	2.002*	[1.103, 3.632]	1.041	[0.553, 1.960]
Woman × Unemployed	0.932	[0.187, 4.643]	1.275	[0.397, 4.096]	0.988	[0.296, 3.297]	3.759	[0.427, 33.071]
Other	3.065	[0.746, 12.591]	1.962	[0.639, 6.203]	2.758	[0.981, 7.752]	2.652	[0.453, 6.027]
Woman × Other	—	—	0.568	[0.056, 5.806]	0.446	[0.053, 3.781]	1.917	[0.075, 48.648]
Unknown	2.334	[0.376, 14.493]	0.740	[0.173, 3.155]	1.769	[0.478, 6.537]	1.029	[0.236, 4.493]
Woman × Unknown	0.978	[0.33, 28.825]	6.037	[0.528, 69.047]	—	—	19.588	[0.928, 413.298]
CJS Involvement (ref: no)								
Yes	0.390**	[0.228, 0.667]	0.140**	[0.075, 0.263]	0.614**	[0.467, 0.807]	0.274**	[0.169, 0.422]
Woman × Yes	2.305	[0.839, 6.331]	1.751	[0.582, 5.266]	1.002	[0.572, 1.755]	0.984	[0.334, 2.900]
Unknown	—	—	0.628	[0.058, 6.779]	1.918	[0.572, 1.599]	3.762	[0.672, 21.058]
Woman × Unknown	—	—	3.091	[0.150, 63.684]	2.406	[0.180, 32.111]	0.415	[0.026, 6.717]
Region (ref: major city)								
Regional/remote	8.362**	[5.625, 12.432]	4.559**	[3.206, 6.477]	1.547**	[1.187, 2.017]	2.220**	[1.563, 3.155]
Woman × Regional/remote	3.820**	[1.507, 9.687]	1.446	[0.787, 2.657]	1.353	[0.837, 2.189]	0.916	[0.460, 1.825]
Unknown	1.530	[0.521, 4.492]	1.920	[0.870, 4.235]	0.957	[0.572, 1.599]	3.762	[0.114, 2.430]
Woman × Unknown	14.467**	[1.648, 127.030]	0.628	[0.058, 6.768]	3.539	[0.979, 12.978]	—	—
Recent injecting (ref: no)								
Yes	0.664*	[0.446, 0.987]	0.204**	[0.143, 0.291]	1.267	[0.988, 1.624]	0.429**	[0.307, 0.601]
Woman × Yes	1.787	[0.806, 3.963]	0.791	[0.417, 1.499]	1.239	[0.774, 1.984]	1.037	[0.531, 2.023]
Unknown	1.505	[0.553, 4.097]	0.758	[0.232, 1.778]	2.286**	[1.242, 4.208]	1.015	[0.455, 2.430]
Woman × Unknown	0.593	[0.103, 3.423]	0.853	[0.246, 2.952]	0.517	[0.174, 1.536]	2.931	[0.895, 9.595]

*Note*: Reference class = *Heroin + lower polysubstance use* (*n* = 1565, 52.3%). All predictors entered in a single step.

Abbreviations: AOR, adjusted odds ratio; CI, confidence interval; CJS, criminal justice system; OAT, opioid agonist treatment; –unable to compute statistics due to small *n*.

* *p* < 0.05.

** *p* < 0.01.

The final model was significant, *X* ^2^(68) = 899.99*, p* < 0.001 and explained 27.8% of the variance in latent class membership. Parameter estimates showed that, compared to the *Heroin + lower polysubstance use class*, people in the *Pharmaceutical + polysubstance use class* were less likely to report ‘other’ living arrangements compared to living with others, less likely to have CJS involvement, more likely to live in regional/remote areas and less likely to report recent injecting use. Gender interactions also demonstrated that women in this class were more likely to have ‘unknown’ living arrangements and either lived in regional/remote areas or had ‘unknown’ region data. People in the *Pharmaceutical + lower polysubstance use class* were more likely to have ‘unknown’ accommodation data, less likely to have ‘other’ living arrangements compared to living with others, less likely to have CJS involvement, more likely to live in regional/remote areas and less likely to report recent injecting use. There were no gender interactions for this class. People in the *Heroin + polysubstance use class* were less likely to be aged 35 or older, more likely to live in unstable accommodation, more likely to be unemployed, less likely to have CJS involvement, more likely to live in regional/remote areas and more likely to have ‘unknown’ injecting drug use data. There were no gender interactions for this class. Finally, people in the *OAT + polysubstance use class* were less likely to have CJS involvement or recent injecting drug use and more likely to live in regional/remote areas. Women living alone were less likely to belong in this class compared to men living alone or women living with others.

## 4 Discussion

Understanding the context that brings clients to treatment is an important aspect of improving the responsivity of residential treatment to complex client needs and presentations and in reducing their risk of post‐treatment overdose. This is in line with AOD Clinical Care standards [57] which emphasise the importance of comprehensive risk assessment and individualised care planning. The current results demonstrate that the co‐occurrence of sociodemographic and polysubstance use risks, including how these risks differ by gender, are important considerations when tailoring treatment to individual client needs and in adequately planning a client's post‐treatment supports.

Results of the LCA revealed distinct classes based on primary opioid and other substance/s of concern. Results showed clear polysubstance use patterns—particularly for primary heroin and primary pharmaceutical opioid users. Likelihood of other substance use in these groups shows a clear demarcation between clients who use substances in addition to their primary drug of concern and clients who use their primary drug of concern in relative isolation. However, consistent with prior research, some level of polysubstance use appeared to be the norm for people accessing residential treatment for opioid use [39]. The generally high likelihood of other opioid and sedative use, particularly, for primary heroin and pharmaceutical opioid users, is concerning given the compounding depressive effects on the central nervous system, which can increase the risk of sedation, supressed breathing, impaired cognitive function and fatal overdose [24]. The generally high use of stimulants across classes is also consistent with global stimulant use rates and common polysubstance use patterns, where stimulants are often used concurrently with opioids to enhance/maintain a ‘high’ or manage undesired effects [58]. In line with residential treatment's focus on intensive care [5], these results highlight the importance of comprehensive risk assessment that examines the entirety of a person's substance use, rather than a singular focus on a person's primary substance of concern. Additionally, only around 20% of participants in the current study accessed residential treatments that prescribe OAT. Given most residential treatments are ‘opioid‐free’ and do not prescribe OAT [25], many clients will leave residential treatment with no opioid tolerance, placing them at increased risk of mortality in the post‐treatment period [26], particularly, when using multiple substances. This under‐coverage or under‐utilisation of residential services that prescribe OAT is concerning given that clients accessing these services also experience a large number of concurrent sociodemographic risk factors (e.g., higher age, unstable accommodation, unemployment, high psychological distress). Expanding coverage of and access to residential services that prescribe OAT is therefore an important area for future research and policy consideration.

Results of the multinomial logistic regression demonstrated some significant class differences in sociodemographic overdose risk, though limited interactions between risk and gender. Compared to the *Heroin + lower polysubstance use class*, people in all other classes were more likely to reside in regional or remote areas. This demonstrates the importance of expanding availability of and access to substance use treatments in regional or remote areas and ensuring the effective assessment and management of polysubstance use risk. It was also observed that women were more likely than men to live in regional/remote areas, but access treatments located in major cities. Limited access to female‐specific services in regional/remote areas, coupled with rural infrastructure problems, insufficient resources and geographic isolation may push women to seek residential treatment further away from their home [59]. This increase in travel burden, as well as limited regional resources for effective continuing care, may therefore be an important post‐treatment consideration for women living in regional/rural areas. Of particular note, the *Heroin + polysubstance use class* was associated with a range of cumulative overdose risks, including being aged under 35+ years, unstable accommodation, being unemployed and living in regional/remote areas, demonstrating important considerations for post‐treatment support for this group. Although older age groups are emerging as a group at increased overdose risk [12, 13], the associations between heroin and polysubstance use and younger age (< 35 years) highlight that this group also requires targeted overdose prevention and early intervention programmes. In addition, people in this class were more likely to have ‘unknown’ injecting drug use data, likely a function of service level practices and procedures regarding collecting lifetime and recent injecting drug use information. The lack of injecting data for this group, particularly given additional cumulative risks, represents an important area for further consideration in order to understand and reduce the potential harms associated with injection methods (e.g., HIV/AIDS, blood–borne viruses). Overall, these results highlight the complex interplay between sociodemographic and polysubstance use risk, all of which influence a client's post‐treatment risk of overdose or opioid‐related harms. These results demonstrate the importance of adequately tailoring a client's treatment to effectively manage risks, as well as the integration of appropriate post‐treatment supports.

Results also demonstrate that many sociodemographic risks appear to be present regardless of the client's gender or polysubstance use profile. This emphasises the importance of comprehensive risk assessment at treatment entry, as outlined in the New South Wales AOD Clinical Care Standards [57] and the provision of post‐treatment support for all clients in areas such as accommodation, employment or financial aid, social support and mental health care. This is consistent with concerns voiced by consumer representatives, who noted a lack of adequate support when transitioning out of residential treatment in areas such as housing, financial insecurity, mental health concerns, family custody issues and general living skills [6]. The similarities in sociodemographic risk profiles may also indicate a lack of gender‐specific risks being captured in routinely collected data. For example, the NADAbase provides opportunities for data collection related to domestic violence and suicide, though these variables are not mandated and are therefore not often reliably collected by treatment services. Research has demonstrated that women who use opioids are more likely to experience interpersonal violence [60, 61, 62], have greater psychiatric comorbidity [63, 64] and higher rates of suicidal ideation and behaviours [33]. This emphasises the importance of these factors when assessing women's overdose risk. Improved guidance on how data collection and reporting can be embedded within service provision, including the implementation of routine outcome monitoring, may also aid in improving reliable risk assessment and data reporting. In addition, the monitoring of client progress and delivery of feedback to clients and clinicians may enhance clinical decision‐making regarding risk management and improve treatment outcomes for clients during their treatment episode.

The current study has several strengths. It utilised a large and specific sample to provide important information regarding opioid users accessing residential treatment in New South Wales, Australia, which has been previously under‐researched. LCA is a valid and rigorous analytical method of identifying unique typologies or classes of participants and this analysis provided important and novel information regarding the specific polysubstance use profiles and risk factors of people who report primary opioid use accessing residential treatment. While such a rigorous analysis may not be feasible in routine practice, these results demonstrate the importance of considering a client's overall substance use profile, rather than a singular focus on primary substance of concern. Utilisation of LCA in large‐scale data‐based studies can also be useful in understanding sub‐populations of people who use AODs, which can inform clinical practice. There are also several limitations of the current study. While LCA is a valid statistical analysis, class assignment is based on probabilities and exact class membership cannot be determined. There is also some level of uncertainty within the results of the multinomial logistic regression as evidenced by wide confidence intervals. While the specific sample allowed for in‐depth analysis of an Australian population of opioid users, the generalisation of these results to other contexts is not certain, particularly as NADA member services are publicly funded. In addition, the lack of reliable data collection across some variables may result in important sociodemographic risks that were overlooked in the current analysis (e.g., prior overdose, sexuality, suicidality). Further, the sometimes large degree of missing/uncertain data (including for measures such as psychological distress or substance dependence severity) may result in an under‐representation of high‐risk clients. While this is similar to the way in which information regarding client risk is known (or not known) in clinical practice, it highlights the need for improved data collection and reporting, at both the service and sector level, so that individual client's risks can be more confidently appraised. The lack of linkage to external data sources also restricts the study to outcomes captured within the NADABase, omitting key factors such as deaths, hospitalisations and social security support, which are important for a more comprehensive understanding of client's substance use and overdose risk. The current sample was also restricted to those who identified as a man or a woman, with cisgender and transgender men and women grouped together (based on self‐identified gender, rather than sex assigned at birth). As such, some additional complexities regarding gender may have been missed. Further research is needed to investigate the diverse and unique needs of people who identify as part of the non‐binary and/or transgender communities. Further longitudinal studies regarding the complex relationships between gender, sociodemographic risk, polysubstance use and overdose may also aid in addressing some of the limitations associated with cross‐sectional research.

## 5 Conclusion

Effective overdose prevention and harm reduction strategies both during and post residential treatment need to consider individual's complex and unique accumulation of risk. Many supports must be provided to all clients during and post‐residential treatment, including supports focused on employment, housing and mental health, as well as general overdose education and harm‐reduction strategies. Additional individualised care and support should also be integrated based on client's unique polysubstance use and sociodemographic risk profiles. Future research may benefit from examining polysubstance use and sociodemographic overdose risk profiles over longer periods of time, including assessing the relationship between risk profiles, mental health and substance use outcomes. Considerations should also be made regarding how data collection and reporting regarding key overdose risk factors and treatment outcomes can be improved within service provision and sector‐level datasets.

## Author Contributions

**Chloe J. Haynes:** conceptualisation, methodology, formal analysis, investigation, writing – original draft, writing – review and editing, visualisation, project administration. **Alison K. Beck:** conceptualisation, methodology, writing – review and editing, supervision. **Peter J. Kelly:** conceptualisation, methodology, writing – review and editing, supervision. **Mei Lin Lee:** data curation, methodology, writing – review and editing. **Robert Stirling:** writing – review and editing. **Suzie Hudson:** writing – review and editing. **Laura Robinson:** methodology, writing – review and editing. **Michele Campbell:** writing – review and editing. **Carolyn Stubley:** writing – review and editing. **Briony Larance:** conceptualisation, methodology, writing – review and editing, supervision.

## Conflicts of Interest

P.J.K. and B.L. hold research consultancies at NADA. M.L.L., R.S., M.C., and C.S. are employees of NADA. C.J.H., A.K.B., S.H. and L.R. have no conflicts of interest to report.

## Supporting information


**Data S1.**Supporting Information.

## Data Availability

The data that support the findings of this study are available from the corresponding author upon reasonable request.
