# Supplementary material for: Gender and Overdose Risk Factors Among Clients Entering Residential Treatment for Opioid Use
Source: Drug Alcohol Rev. 2025 Jul 7;44(6):1786–804. doi: 10.1111/dar.70004 (PMC12405814; doi:10.1111/dar.70004)
Supplement: Supplementary file 1 — Data S1.Supporting Information. [file DAR-44-1786-s001.docx]

**Gender and overdose risk factors among clients entering residential treatment for opioid use**

**(Running title: Gender and opioid overdose risk) – Supplementary Material**

Chloe J. Haynes^1*^, Alison K. Beck^1^, Peter J. Kelly^1^, Mei Lin Lee^2^, Robert Stirling^2, 3^, Suzie Hudson^4, 5^, Laura Robinson^1^, Michele Campbell^2, 7^, Carolyn Stubley^2, 6^, Briony Larance^1^

^1^School of Psychology, University of Wollongong, Australia.

^2^Network of Alcohol and Other Drug Agencies (NADA), Sydney, Australia.

^3^Drug Policy Modelling Program, Social Policy Research Centre, UNSW, Sydney, Australia.

^4^Centre for Alcohol and Other Drugs, NSW Ministry of Health, Australia.

^5^National Drug & Alcohol Research Centre, UNSW, Sydney, Australia.

^6^We Help Ourselves (WHOS), Sydney, Australia.

^7^School of Psychology, University of Queensland, Australia.

^*^ Corresponding author at: University of Wollongong, Northfields Avenue, Wollongong, NSW, Australia, 2500. *Email address:* cjh893@uowmail.edu.au

Funding: CH was supported by an Australian Government Research Training Program (AGRTP) scholarship while conducting the research. This work was also supported by the National Health and Medical Research Council (NHMRC) Meaningful Outcomes in Substance Use Treatment Centre of Research Excellence.

Each author certifies that their contribution to this work meets the standards of the International Committee of Medical Journal Editors.

**Appendix A.**

STROBE Statement—Checklist of items that should be included in reports of *cross-sectional* *studies*

|  | Item No | Recommendation |  |  |
| --- | --- | --- | --- | --- |
| **Title and abstract** | 1 | (*a*) Indicate the study’s design with a commonly used term in the title or the abstract |  |  |
|  |  | (*b*) Provide in the abstract an informative and balanced summary of what was done and what was found |  |  |
| Introduction | | |  |  |
| Background/rationale | 2 | Explain the scientific background and rationale for the investigation being reported |  |  |
| Objectives | 3 | State specific objectives, including any prespecified hypotheses |  |  |
| Methods | | |  |  |
| Study design | 4 | Present key elements of study design early in the paper |  |  |
| Setting | 5 | Describe the setting, locations, and relevant dates, including periods of recruitment, exposure, follow-up, and data collection |  |  |
| Participants | 6 | (*a*) Give the eligibility criteria, and the sources and methods of selection of participants |  |  |
| Variables | 7 | Clearly define all outcomes, exposures, predictors, potential confounders, and effect modifiers. Give diagnostic criteria, if applicable |  |  |
| Data sources/ measurement | 8* | For each variable of interest, give sources of data and details of methods of assessment (measurement). Describe comparability of assessment methods if there is more than one group |  |  |
| Bias | 9 | Describe any efforts to address potential sources of bias |  |  |
| Study size | 10 | Explain how the study size was arrived at |  |  |
| Quantitative variables | 11 | Explain how quantitative variables were handled in the analyses. If applicable, describe which groupings were chosen and why |  |  |
| Statistical methods | 12 | (*a*) Describe all statistical methods, including those used to control for confounding |  |  |
|  |  | (*b*) Describe any methods used to examine subgroups and interactions |  |  |
|  |  | (*c*) Explain how missing data were addressed |  |  |
|  |  | (*d*) If applicable, describe analytical methods taking account of sampling strategy |  |  |
|  |  | (*e*) Describe any sensitivity analyses |  |  |
| Results | | |  |  |
| Participants | 13* | (a) Report numbers of individuals at each stage of study—eg numbers potentially eligible, examined for eligibility, confirmed eligible, included in the study, completing follow-up, and analysed |  |  |
|  |  | (b) Give reasons for non-participation at each stage |  |  |
|  |  | (c) Consider use of a flow diagram |  |  |
| Descriptive data | 14* | (a) Give characteristics of study participants (eg demographic, clinical, social) and information on exposures and potential confounders |  |  |
|  |  | (b) Indicate number of participants with missing data for each variable of interest |  |  |
| Outcome data | 15* | Report numbers of outcome events or summary measures |  |  |
| Main results | 16 | (*a*) Give unadjusted estimates and, if applicable, confounder-adjusted estimates and their precision (eg, 95% confidence interval). Make clear which confounders were adjusted for and why they were included |  |  |
|  |  | (*b*) Report category boundaries when continuous variables were categorized |  |  |
|  |  | (*c*) If relevant, consider translating estimates of relative risk into absolute risk for a meaningful time period |  |  |
| Other analyses | 17 | Report other analyses done—eg analyses of subgroups and interactions, and sensitivity analyses |  |  |
| Discussion | | |  |  |
| Key results | 18 | Summarise key results with reference to study objectives |  |  |
| Limitations | 19 | Discuss limitations of the study, taking into account sources of potential bias or imprecision. Discuss both direction and magnitude of any potential bias |  |  |
| Interpretation | 20 | Give a cautious overall interpretation of results considering objectives, limitations, multiplicity of analyses, results from similar studies, and other relevant evidence |  |  |
| Generalisability | 21 | Discuss the generalisability (external validity) of the study results |  |  |
| Other information | | |  |  |
| Funding | 22 | Give the source of funding and the role of the funders for the present study and, if applicable, for the original study on which the present article is based |  |  |

*Give information separately for exposed and unexposed groups.

**Note:** An Explanation and Elaboration article discusses each checklist item and gives methodological background and published examples of transparent reporting. The STROBE checklist is best used in conjunction with this article (freely available on the Web sites of PLoS Medicine at http://www.plosmedicine.org/, Annals of Internal Medicine at http://www.annals.org/, and Epidemiology at http://www.epidem.com/). Information on the STROBE Initiative is available at www.strobe-statement.org.

| Appendix B.  Original data coding in NADABase and recoding for analysis. | | | |
| --- | --- | --- | --- |
| Variable | **Original coding in NADAbase** | **Recoding for demographic analysis** | **Recoding into risk factors** |
| Age | Date of birth (DD/MM/YYYY) | Calculation of age as of August 2023 and age (integer).  Age (integer) then coded into 18-29 years; 30-39 years; 40-49 years; 50-59 years; 60+ years. | Aged under 35; aged 35 or over. |
| Country of Birth | Four digit country of birth code based on Standard Australian Classification of Countries (SACC) (SACC; 1). | United Nations Geoscheme (2): first recoded based on sub-regions, some categories collapsed due to small numbers.  Final groupings: Australia, New Zealand, Asia, Europe, Northern America, Latin America and the Caribbean, Northern Africa, Sub-Saharan Africa, Other Oceania (including Micronesia, Melanesia, Polynesia), Other, Unknown/ inadequately described. | N/A – limited countries of birth outside of Australia and New Zealand, limited empirical support regarding country of birth as risk factor for overdose. |
| Sexuality | Straight or heterosexual; lesbian, gay, homosexual; bisexual; queer; another term; unknown; prefer not to answer; not stated or inadequately described. | Straight/heterosexual; LGBTQIA+ (including lesbian, gay, homosexual; bisexual; queer; another term); unknown or inadequately described (including prefer not to answer). | N/A – limited data available. |
| Accommodation | Rented house or flat (public or private); privately owned house or flat; boarding house; hostel/supported accommodation; psychiatric hospital; alcohol/other drug treatment residence; shelter/refuge; prison/detention centre; caravan on a serviced site; no usual residence/ homeless; other; not known. | Stable accommodation (including rented or privately owned house or fat), unstable/temporary accommodation (including boarding house; hostel/supported accommodation; psychiatric hospital; alcohol and other drug treatment residence; shelter/refuge; caravan on a serviced site); prison/detention centre; homeless/no usual residence; other/inadequately described. | Stable accommodation; unstable accommodation (including homeless/no usual residence); other; unknown (including not stated, inadequately described) |
| Living arrangements | Alone; spouse or partner; single parent with child(ren); spouse or partner and child(ren); parent(s); other relative(s); friend(s); friend(s) or parent(s) or relative(s) and child(ren); other; not known or not stated or inadequately described. | Used to code two separate variables:   1. Living arrangements: alone; spouse or partner; parents/friends/relatives; other/ inadequately described. 2. Living with dependent children (yes): single parent with child(ren), spouse or partner and child(ren), friend(s) or parent(s) or relative(s) and child(ren). | Living alone; living with others (including spouse or partner; parents/friends/ relatives and children); other; unknown (including not stated, inadequately described) |
| Source of income | Full-time employment; part-time employment; temporary benefit (e.g., unemployment); pension (e.g., aged, disability); student allowance; dependent on others; retirement fund; no income; other; not stated/not known or inadequately described. | Full or part time employment; temporary government benefits (including temporary benefit; student allowance); permanent government benefits (including pension); no income (including dependent on others); other/ inadequately described (including retirement fund). | Employed; unemployed (including temporary benefits, permanent benefits, no income, other/inadequately described); other; unknown (including not stated, inadequately described) |
| Location of usual residence | Postcode of client’s usual residence. | Recoded based on Remoteness Structure (3): Major Cities; Inner Regional; Outer Regional; Remote; Very Remote.  Collapsed into Major Cities; Regional; Remote; Unknown. | Major city, regional/rural, unknown (including not stated, inadequately described) |
| Primary opioid of concern | Coding for principal drug of concern according to Australian Standard Classification of Drugs of Concern (4). | Recoded into principal opioid of concern:  heroin; oxycodone; codeine; morphine; fentanyl; methadone; buprenorphine; other opioid analgesic (including levomethadyl acetate hydrochloride, meperidine analogues, pethidine, tramadol, and any other pharmaceutical opioids or organic, semisynthetic or synthetic opiate analgesics not further defined). | Primary heroin use (yes/no); primary oxycodone use (yes/no); primary codeine use (yes/no); primary morphine use (yes/no); primary fentanyl use (yes/no); primary methadone use (yes/no); primary buprenorphine use (yes/no); primary other opioid use (yes/no). |
| Other substance(s) of concern | Coding for other drug of concern according to Australian Standard Classification of Drugs of Concern (4).  NADABase provides opportunity to specify up to 15 other substances of concern. | First 5 other substances of concern recoded into Other Drug (1-5): not stated/inadequately described; opioids; gambling; non-opioid analgesics; alcohol; anaesthetics; barbiturates; benzodiazepines; GHB; other sedatives & hypnotics; amphetamines; ephedra alkaloids; hallucinogens; caffeine; cocaine; methylphenidate; nicotine; steroids; antidepressants and antipsychotics; inhalants; cannabinoids; others.  Collapsed into opioids; alcohol; benzodiazepines or sedatives (including anaesthetics; barbiturates, benzodiazepines; GHB; other sedatives or hypnotics); cannabinoids; stimulants (including amphetamines; ephedra alkaloids; cocaine; methylphenidate); other (including gambling; non-opioid analgesics; hallucinogens; caffeine; nicotine; steroids; antidepressants/ antipsychotics; inhalants; other).  Composite variable computed based on whether client identified each substance across ANY of the 5 other drug categories. | Other opioid use (yes/no); other alcohol use (yes/no); other benzodiazepine or sedative use (yes/no); other cannabinoid use (yes/no); other stimulant use (yes/no); other substance use (yes/no). |
| Injecting use | Not collected; last injected within previous 3 months; last injected more than 3 months but less than 12 months ago; last injected 12 months ago or more; never injected; not stated/inadequately described. | Lifetime injecting use: yes; no (including not collected or not stated/inadequately described). | Recent injecting use: yes (last injected within previous 3 months), n; unknown (including not stated, inadequately described, not collected) |
| Source of referral | Self; family member/friend; general practitioner; medical officer/specialist; psychiatric hospital; other hospital; residential community mental health care unit; residential alcohol and other drug treatment agency; other residential community care unit; education institution; non-residential community mental health centre; non-residential alcohol and other drug treatment agency; non-residential community health centre; other non-health service agency; police diversion; court diversion; other criminal justice setting; workplace (EAP); family and child protection service; needle and syringe program; medically supervised injecting centre; other; not stated/inadequately described | Self; family member/friend; medical professional/hospital (including general practitioner; medical officer/specialist; psychiatric hospital; other hospital); residential community care unit/agency; non-residential community care unit/agency; other non-health service; police/court diversion or justice system; family and child protective services; other (including educational institution; workplace; needle and syringe programs; medically supervised injection centres; other); not stated/inadequately described. | Criminal justice involvement (police/criminal justice referral AND/OR living in prison from accommodation variable) – yes/no |
| Main service | Counselling; withdrawal management (detoxification); rehabilitation activities; pharmacotherapy; support and case management only; assessment only; information and education only; other | Counselling; withdrawal management; rehabilitation; support and case management; assessment only; other (including pharmacotherapy; information and education only; detoxification). | N/A – limited evidence to suggest relationship between service and overdose |
| Location of service | Postcode of agency. | Recoded based on Remoteness Structure (3): Major Cities; Inner Regional; Outer Regional; Remote; Very Remote.  Collapsed into Major Cities; Regional; Remote; Unknown. | N/A – client’s location used as primary measure of location |

| Appendix C.  Support for coding of sociodemographic factors into overdose risk factors. | | |
| --- | --- | --- |
| Variable | **Evidence for risk factor** | **Clinical relevance** |
|  |  |  |
| Gender  Male  Female | Though opioid deaths are more common among males (5-7), women’s overdose risk, and the accumulation of polysubstance and sociodemographic risks for women, is currently under-researched. Women also present to treatment with a larger number of comorbidities that may impact substance use and overdose risk. | Important to consider gender in treatment planning and provision – differences in presentation, risk factors, and treatment needs. |
| Age  Younger (18-34)  Middle aged to older (35+) | Shift in age of opioid-induced deaths from younger age group (15-34) to middle aged and older groups (35-55 and 55+) (5, 6). | Identify need to provide extra age-appropriate post-treatment support, particularly for older clients. |
| Accommodation  Stable  Unstable (including homeless, prison, or unknown) | Reciprocal relationship between homelessness/unstable accommodation and drug use (8, 9).  Increased risk of overdose associated with low socioeconomic status (6). | Identify need to provide extra support post-treatment regarding housing stability. |
| Living arrangements  With others  Alone | 78% of drug-related deaths from 1997 to 2020 occurred in the home (5). | Identify need to link client in with extra supports outside of the home post-treatment, ensuring regular check ins if the person is living alone. |
| Employment  Employed  Unemployed | Increased risk of overdose associated with low socioeconomic status generally (6) and with unemployment or job loss (10). | Identify need to provide extra support post-treatment regarding employment/financial aid. |
| Criminal justice involvement  No  Yes (residing in prison and/or CJS referral to treatment) | People who have spent time in prison are at a higher risk of overdose in the community post-release (11, 12).  Other criminal justice involvement including arrests, drug or misdemeanour charges also associated with higher risk of overdose (13). | Identify need to provide extra post-treatment supports for those with criminal justice involvement, providing links to specific supports post-incarceration or criminal justice proceedings. |
| Region  Major Cities  Regional/Remote | Though rates of drug-induced deaths are higher in major cities (5), clients in regional/remote areas experience compounding risks due to inadequate service coverage, rural infrastructure problems, lack of access/availability of other supports, and geographic isolation (14). | Identify need to provide specialised post-treatment supports based on location of client and location of accessible services. |
| Recent injecting drug use (within previous 3 months)  No  Yes | Unsafe injecting practices and regular injecting associated with high rates of overdose (8, 15). | Identify need to provide education and support around safe injecting practices. |
| Primary opioid of concern  Heroin  Oxycodone  Codeine  Morphine  Fentanyl  Methadone  Buprenorphine  Other opioid analgesic | Natural and semi-synthetic opioids (e.g., morphine, oxycodone) most common opioid in opioid-related deaths, followed by heroin (5).  Up-scheduling of over-the-counter codeine products reduced poisoning calls and emergency department presentations (16), but often associated with accidental overdose and mixed-drug toxicity (17).  Increasing rates of fentanyl use and fentanyl-related deaths (18).  Also increase in methadone-related deaths (18). | Importance of tailoring post-treatment supports based on client’s individualised and specific substance use patterns and risk. |
| Other substance(s) of concern^6^  Another opioid  Alcohol  Benzodiazepines or sedatives^7^  Cannabinoids  Stimulants^8^  Other substance^9^ | Majority of drug overdose deaths include two or more drug classes (5, 6).  Mixing opioids and any other substance increases risk of overdose due to 1) cumulation of depressant effects or 2) depressant + stimulant effects masking effects of each drug and leading to increased consumption of one or both substance(s). | Importance of tailoring post-treatment supports based on client’s individualised and specific substance use patterns and risk. |

| **Appendix D.**  Probability of reporting primary opioid and other substance use by class (%). | | | | | |
| --- | --- | --- | --- | --- | --- |
| **Probability of specific substance use** | **Latent Class** | | | | |
|  | **Pharmaceutical + polysubstance use (n=202, 6.7%)** | **Pharmaceutical + lower polysubstance use**  **(n=303, 10.1%)** | **Heroin + polysubstance use (n=665, 22.2%)** | **Heroin + lower polysubstance use (n=1565, 52.3%)** | **OAT + polysubstance use (n=259,**  **8.7%)** |
| Heroin | 0.0 | 0.0 | **100.0** | **100.0** | 0.0 |
| Pharmaceutical opioid | **100.0** | **100.0** | 0.00 | 0.0 | 0.0 |
| Methadone/  Buprenorphine | 0.0 | 0.0 | 0.00 | 0.0 | **100.0** |
| Another Opioid | **46.6** | 9.6 | 34.4 | 4.8 | 27.4 |
| Alcohol | 19.8 | 10.0 | **28.8** | 3.0 | 14.7 |
| Sedatives | 21.7 | 7.1 | **30.2** | 2.7 | 15.1 |
| Cannabis | **55.4** | 4.6 | 40.4 | 5.4 | 25.5 |
| Stimulants | **45.7** | 8.2 | 44.9 | 18.3 | 20.8 |
| Other Substance | **38.2** | 11.9 | 25.5 | 5.7 | 18.5 |

1. Australian Bureau of Statistics. Standard Australian Classification of Countries (SACC): Australian Bureau of Statistics; 2016 [Available from: <https://www.abs.gov.au/statistics/classifications/standard-australian-classification-countries-sacc/latest-release>.

2. United Nations. Methodology: Standard country or area codes for statistical use (M49): Department of Economics and Social Affairs Statistics Division; 2024 [Available from: <https://unstats.un.org/unsd/methodology/m49/>.

3. Australian Bureau of Statistics. Remoteness Structure: Australian Statistical Geography Standard (ASGS) Edition 3: Australian Bureau of Statistics; 2021 [Available from: <https://www.abs.gov.au/statistics/standards/australian-statistical-geography-standard-asgs-edition-3/jul2021-jun2026/remoteness-structure>.

4. Australian Bureau of Statistics. Australian Standard Classification of Drugs of Concern: Australian Bureau of Statistics; 2011 [Available from: <https://www.abs.gov.au/statistics/classifications/australian-standard-classification-drugs-concern/latest-release#coding-index>.

5. Chrzanowska A, Man N, Akhurst J, Sutherland R, Degenhardt L, Peacock A. Trends in overdose and other drug-induced deaths in Australia, 2002-2021. Sydney: National Drug and Alcohol Research Centre, UNSW Sydney; 2023.

6. Australian Bureau of Statistics. Opioid-induced deaths in Australia 2019 [Available from: <https://www.abs.gov.au/articles/opioid-induced-deaths-australia>.

7. Butelman ER, Huang Y, Epstein DH, Shaham Y, Goldstein RZ, Volkow ND, et al. Overdose mortality rates for opioids or stimulants are higher in males than females, controlling for rates of drug misuse: State-level data. medRxiv. 2023.

8. Australian Institute of Health and Welfare. Alcohol, tobacco & other drugs in Australia 2022 [Available from: <https://www.aihw.gov.au/reports/alcohol/alcohol-tobacco-other-drugs-australia/contents/drug-types/illicit-opioids-including-heroin>.

9. Penington Institute. Breaking the cycle - Opioid dependence and housing stability - Final report Australia: Penington Institute; 2020.

10. Azagba S, Shan L, Qeadan F, Wolfson M. Unemployment rate, opioids misuse and other substance abuse: quasi-experimental evidence from treatment admissions data. BMC Psychiatry. 2021;21(1):22.

11. Degenhardt L, Larney S, Kimber J, Gisev N, Farrell M, Dobbins T, et al. The impact of opioid substitution therapy on mortality post-release from prison: retrospective data linkage study. Addiction. 2014;109(8):1306-17.

12. Merrall EL, Kariminia A, Binswanger IA, Hobbs MS, Farrell M, Marsden J, et al. Meta-analysis of drug-related deaths soon after release from prison. Addiction. 2010;105(9):1545-54.

13. Krawczyk N, Schneider KE, Eisenberg MD, Richards TM, Ferris L, Mojtabai R, et al. Opioid overdose death following criminal justice involvement: Linking statewide corrections and hospital databases to detect individuals at highest risk. Drug and Alcohol Dependence. 2020;213:107997.

14. Passey M, Sheldrake M, Leitch K, Gilmore V. Impact of case management on rural women's quality of life and substance use. Rural and Remote Health. 2007;7(710).

15. Geddes L, Iversen J, Darke S, Dietze P, Maher L. Prevalence and correlates of multiple non-fatal opioid overdoses among people who inject drugs who utilise needle syringe programs in Australia. International Journal of Drug Policy. 2021;96:103245.

16. Bishop M, Schumann JL, Gerostamoulos D, Wong A. The impact of codeine upscheduling on overdoses, Emergency Department presentations and mortality in Victoria, Australia. Drug Alcohol Depend. 2021;226:108837.

17. Roxburgh A, Hall WD, Burns L, Pilgrim J, Saar E, Nielsen S, et al. Trends and characteristics of accidental and intentional codeine overdose deaths in Australia. Medical Journal of Australia. 2015;203(7):299-.

18. Penington Institute. Australia’s Annual Overdose Report 2023. Melbourne: Penington Institute; 2023.
